# Use, timing and outcome of coronary angiography in patients with high-risk non-ST-segment elevation acute coronary syndrome in daily clinical practice: insights from a ‘real world’ prospective registry

**DOI:** 10.1007/s12471-018-1212-3

**Published:** 2018-12-13

**Authors:** E. A. Badings, R. S. Hermanides, A. Van Der Sluis, J. H. E. Dambrink, A. T. M. Gosselink, E. Kedhi, J. P. Ottervanger, V. Roolvink, W. S. Remkes, E. van’t Riet, H. Suryapranata, A. W. J. van’t Hof

**Affiliations:** 1 Department of Cardiology, Deventer Hospital, Deventer, The Netherlands; 2 Isala Heart Centre, Zwolle, The Netherlands; 3 Teaching Hospital, Deventer Hospital, Deventer, The Netherlands; 4 Department of Cardiology, Radboud University Medical Centre, Nijmegen, The Netherlands; 5 Department of Cardiology, Maastricht UMC+, Maastricht, The Netherlands

**Keywords:** Clinical outcome, Coronary angiography, Delayed invasive strategy, Early invasive strategy, Non-ST-elevation acute coronary syndrome

## Abstract

**Background:**

An early invasive strategy (EIS) is recommended in high-risk patients with non-ST-elevation acute coronary syndrome (NSTE-ACS), defined as coronary angiography (CAG), within 24 h of admission. The aim of the present study is to investigate guideline adherence, patient characteristics associated with timing of the intervention and clinical outcome.

**Methods:**

In a prospective registry, the use and timing of CAG and the characteristics and clinical outcome associated with timing were evaluated in high-risk ACS patients. The outcome of early versus delayed invasive strategy (DIS) was compared.

**Results:**

Between 2006 and 2014, 2,299 high-risk NSTE-ACS patients were included. The use of CAG increased from 77% in 2006 to 90% in 2014 (*p* trend <0.001) together with a decrease of median time to CAG from 23.3 to 14.5 h (*p* trend <0.001) and an increase of patients undergoing EIS from 50 to 60% (*p* trend = 0.002). Patient factors independently related to DIS were higher GRACE risk score, higher age and the presence of comorbidities. No difference was found in incidence of mortality, reinfarction or bleeding at 30-day follow-up. All-cause mortality at 1‑year follow-up was 4.1% vs 7.0% in EIS and DIS respectively (hazard ratio 1.67, 95% confidence interval 1.12–2.49) but was comparable after adjustment for confounding factors.

**Conclusion:**

The percentage of high-risk NSTE-ACS patients undergoing CAG and EIS has increased in the last decade. In contrast to the guidelines, patients with a higher risk profile are less likely to undergo EIS. However, no difference in outcome after 30 days and 1 year was found after multivariate adjustment for this higher risk.

## What’s new?

- For high-risk patients with non-ST-elevation acute coronary syndrome, guidelines recommend early invasive treatment (coronary angiography within 24 h of admission), but only 60% of patients are treated this way in clinical practice.
- In contrast to the guidelines, patients with a higher risk profile are less likely to undergo early invasive treatment.
- No difference in outcome was found between early and late invasive treatment at 30 days and 1 year following multivariate adjustment for risk factors.

## Background

In recent decades, numerous randomised clinical trials have been performed to investigate the optimal timing of intervention in patients with non-ST-elevation acute coronary syndrome (NSTE-ACS). The results of these studies have been summarised in several meta-analyses [1–7]. In summary, earlier intervention showed no significant difference in mortality or reinfarction but only a reduction in the incidence of refractory ischaemia and in duration of hospital stay.

A pre-specified subgroup analysis of the largest trial, TIMACS [8], showed a statistically significant reduction of 35% in the combined endpoint of death, myocardial infarction, and stroke with an early invasive treatment strategy in patients with a Global Registry of Acute Coronary Events (GRACE) risk score of more than 140. Based on the findings of this otherwise negative trial, current guidelines [9] recommend that the timing of angiography be guided by individual risk stratification. An early invasive treatment strategy is recommended in patients with at least one of the following high-risk factors: rise or fall in cardiac troponin, dynamic ST- or T‑wave changes and GRACE risk score >140. This treatment strategy is defined as coronary angiography performed within 24 h of hospital admission.

The aim of the present study is to investigate to what extent these guidelines are followed in clinical practice in patients hospitalised with a NSTE-ACS and at least one high-risk criterion. The application of early and delayed invasive treatment and the association between patient characteristics and the timing of invasive treatment were investigated. In addition, clinical outcome at 30-day and 1‑year follow-up was compared between early and delayed invasive strategy.

## Methods

The BAMI (Dutch abbreviation for ‘Treatment of Acute Myocardial Ischaemia’) registry is a database with all consecutive patients hospitalised with an acute coronary syndrome (ACS) at Isala, a large, non-academic hospital with 24/7 interventional cardiology facilities in Zwolle, the Netherlands. For the present study, we selected patients hospitalised with NSTE-ACS between 2006 and 2014 with at least one high-risk criterion (rise or fall in cardiac troponin compatible with myocardial infarction, dynamic ST- or T‑wave changes and GRACE risk score >140) but without very-high-risk criteria. Patients referred from non-interventional hospitals were excluded.

The rate of patients undergoing coronary angiography and the time between hospitalisation and the start of coronary angiography was calculated and compared over the years. Based on timing, patients were divided into two groups: those undergoing early invasive treatment (coronary angiography performed within 24 h after hospitalisation) and those undergoing delayed invasive treatment (angiography more than 24 h after admission).

Clinical, demographic and procedural characteristics were prospectively collected and compared between the two groups as well as all-cause mortality, reinfarction and bleeding events within 30 days of hospitalisation. All-cause mortality after 1 year was investigated by consulting the Dutch Municipal Personal Records Database of the last known residence of the patient.

Definitions of myocardial infarction were in accordance with the most recent universal definitions [10]. Bleeding events at 30-day follow-up were defined as clinically overt bleeding with a drop in haemoglobin level of at least 2 mmol/l or a blood transfusion of 2 units of packed cells or more. Cut-off values for cardiac enzymes changed over time. Until February 2011, a troponin T level of 0.1 ng/ml was considered to be elevated. After this date, a high-sensitivity troponin assay was used with a cut-off value of 0.014 ng/l. Reference values for creatine kinase (CK) and CK-MB remained stable during the study with cut-off points for CK of 200 U/l (men) and 170 U/l (women) and for CK-MB of 24 U/l or 6% of CK if CK >200 U/l.

### Statistical analysis

Continuous variables are presented as means and standard deviation if normally distributed, otherwise as medians with 25th and 75th percentiles. Categorical variables are presented as percentages. A graph with timing of coronary angiography over time was constructed. Comparison of continuous variables in patients receiving early or delayed invasive angiography were performed by independent samples *t*-test or ANOVA if normally distributed (and after log transformation, if necessary) or Kruskal-Wallis test. For categorical data, the χ^2^ test was used. Multivariate logistic regression analysis was used to identify patient factors independently related to timing of angiography. The outcome of an early and delayed treatment strategy was compared calculating hazard ratios using Cox regression analysis for total mortality, reinfarction and bleeding events. Determinants which affected the regression coefficient of the association between treatment strategy and outcome with more than 10%, were added to the multivariate regression model. Furthermore, hazard ratios were calculated adjusted for GRACE risk score, a validated predictor of adverse cardiovascular events after ACS [11]. A two-sided *p*-value <0.05 was considered statistically significant. Statistical analysis was performed with IBM SPSS version 22.0 (IBM Corp, New York, NY, USA).

## Results

### Patients

**Fig. 1:**
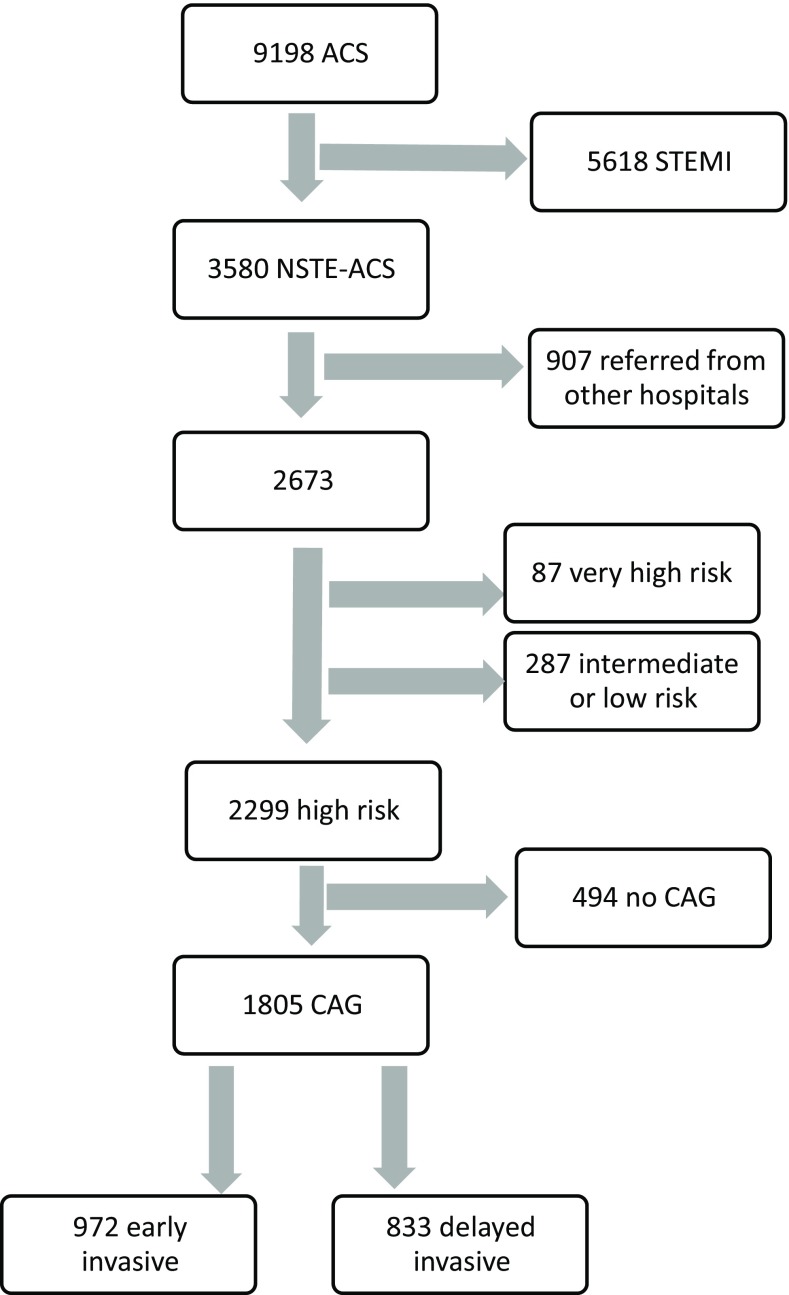
Flowchart of selection of patients with high-risk NSTE-ACS from the BAMI registry (*CAG* coronary angiography, *NSTE-ACS* non-ST-elevation acute coronary syndrome, *STEMI* ST-elevation myocardial infarction)

Between January 2006 and December 2014, 9,198 consecutive patients with a discharge diagnosis of ACS were enrolled in the BAMI registry. In Fig. 1, the flow chart is shown. For the current study, 3,580 patients with a NSTE-ACS were included, of whom 2,673 were hospitalised directly at Isala. In 2,299 patients at least one high-risk and no very-high-risk criterion was present and 1,805 of them underwent coronary angiography. The 494 high-risk patients that did not undergo angiography were older, more often female and median age, GRACE risk score, Killip class as well as the percentage with comorbidities and renal dysfunction were significantly higher.

### Temporal trends in use of angiography

**Fig. 2:**
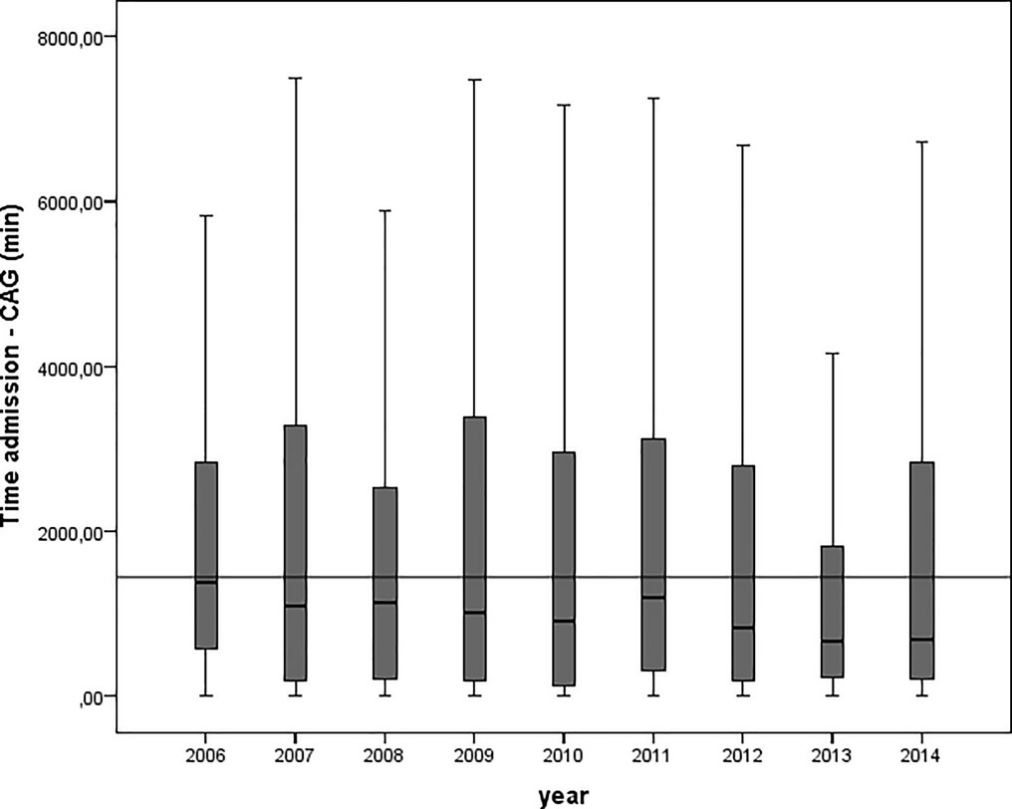
Box and whiskers plot of time in minutes from hospitalisation to coronary angiography. *Horizontal line* depicts 1,440 min (24 h). *Boxes* depict median and 25th and 75th percentiles, whiskers 5th and 95th percentiles (*CAG* coronary angiography, *min* minutes)

The percentage of patients with NSTE-ACS and at least one high-risk criterion that underwent coronary angiography increased gradually from about 77% in 2006 to 90% in 2014 (*p* trend < 0.001). Median time from admission to angiography decreased from 1,400 to 870 min (23.3 to 14.5 h) over the years (*p* < 0.001 analysis of variance, after log transformation, Fig. 2). The proportion of patients undergoing early invasive treatment increased from about 50% in 2006 to 60% in 2014 (*p* trend = 0.002).

### Timing of coronary angiography

In 972 of 1,805 high-risk NSTE-ACS patients (53.9%) coronary angiography was performed within 24 h of hospitalisation. Demographic, clinical and procedural characteristics are shown in Tab. 1. Patients in the delayed treatment group were significantly older and the percentage of patients with diabetes, family and personal history of cardiovascular disease was higher. GRACE risk score, percentages of patients with positive biomarkers at hospitalisation, Killip class >1 and CK above median were also higher in this group. Among the early-treated patients, a higher number with an ST deviation >0.5 mm on admission ECG were seen. Of the patients hospitalised at weekends, 56% underwent angiography after more than 24 h, compared to 47% of those admitted on week days (*p* < 0.001) with a median time from hospitalisation to angiography of 36 versus 21.6 h (*p* = 0.07, after logarithmic transformation).

**Table 1:** Demographic, clinical and procedural characteristics of patients undergoing early (<24 h) or delayed invasive treatment strategy (>24 h after hospitalisation)

	All	Treatment strategy		*p*-value
		Early	Delayed	
*n* (%)	1,805	972 (53.9)	833 (46.1)	–
Time admission-angiography min (median Q1–3)	1,330 (360–3,457)	406 (209–940)	4,008 (2,436–6,723)	–
*Demographics*				
Male gender (%)	68.1	69.1	67.0	0.352
Age (years; mean, SD)	66.8 (12.1)	65.0 (12.2)	68.9 (11.7)	<0.001
*Medical history (%)*				
Diabetes	20.6	16.8	25.2	<0.001
Smoking	31.0	33.2	28.5	0.032
Hypercholesterolaemia	34.1	33.2	35.2	0.366
Positive family history	38.4	40.6	35.8	0.035
Previous MI	17.2	13.9	21.1	<0.001
Previous PCI	18.0	14.5	22.0	<0.001
Previous CABG	12.4	9.4	15.8	<0.001
Previous CVA	3.6	2.8	4.5	0.060
Hypertension	55.0	50.3	60.4	<0.001
*Admission parameters*				
Elevated cardiac enzymes (%)	93.8	92.6	95.2	0.023
GRACE risk score >140^a^ (%)	37.0	32.5	42.1	<0.001
ST segment deviation >0.5 mm (%)	42.4	47.6	36.4	<0.001
CK-max < 24 (umol/l; median, SD)	168 (100–347)	190 (108–425)	146 (92–250)	<0.001
GRACE score^a^ (median, Q1–3)	130 (109–155)	126 (105–150)	135 (112–163)	<0.001
Killip class > 1 (%)	27.7	26.4	29.2	0.175
Creatinine > median (%)	51.0	47.7	54.9	0.003
*Vessel disease (%)*				<0.001
0	11.6	9.3	14.2	–
1	30.4	33.2	27.2	–
>1	58.0	57.5	58.6	–
*Treatment*				<0.001
Conservative (%)	22.6	17.7	28.5	–
CABG performed (%)	18.2	18.0	18.5	–
PCI performed (%)	59.2	64.3	53.1	–
Hospitalisation weekend (%)	26.3	23.0	30.2	0.001
*Medication before angiography*				
ASA (%)	81.2	85.3	76.4	<0.001
Clopidogrel (%)	88.6	91.9	85.0	0.488
G2b3a inhibitor (%)	16.9	22.1	10.9	<0.001
Heparin (%)	63.7	68.9	57.7	<0.001
*At discharge *(%)				
ACE-I (%)	59.0	60.6	57.2	0.159
AII blockers (%)	11.8	9.3	14.6	0.001
RAS inhibition^b^ (%)	70.7	70.8	70.6	0.926
ASA (%)	83.1	86.2	79.6	<0.001
Beta blockers (%)	91.3	91.1	91.6	0.742
Calcium antagonist (%)	20.8	17.1	25.0	<0.001
P2Y_12_ inhibitor (%)	70.7	72.6	68.5	0.055
Coumarin (%)	17.5	12.1	23.5	<0.001
Nitrate (%)	18.0	12.3	24.5	<0.001
Statin (%)	85.0	85.1	84.8	0.842

*AII* angiotensin II, *ACE-I* angiotensin converting enzyme inhibitor, *ASA* acetylsalicylic acid, *CABG* coronary arterial bypass grafting, *CK* creatine kinase, *CVA* cerebrovascular accident, *GRACE* Global Registry of Acute Coronary Events, *MI* myocardial infarction, *PTCA* percutaneous transluminal coronary angioplasty, *Q* quartile, *RAS* renin angiotensin system, *SD* standard deviation

^a^Based on less than 90% of patients

^b^ACE-I and/or AII blockers

Significantly more patients in the early invasive treatment group were treated with anti-thrombotics and anti-coagulants before angiography and underwent a percutaneous coronary intervention (PCI). The percentages of patients undergoing coronary artery bypass graft (CABG) were comparable. Discharge medication differed in the prescription of acetylsalicylic acid (more often in the early group) and coumarins, calcium blockers and nitrates (more often in the delayed group). The use of beta blockers, RAS blockers and P2Y_12_ inhibitors was comparable. Factors independently related to delayed angiography are shown in Tab. 2 and include higher age, absence of ST-segment deviation >0.5 mm, history of hypertension, diabetes, a previous PCI or CABG as well as hospitalisation at weekends. Patients that were included in the registry earlier were more likely to undergo delayed angiography.

**Table 2:** Multivariate regression analysis of factors independently related with timing of coronary angiography (odds ratio of delayed versus early angiography)

	OR	95% CI	*p*-value
Age (per year)	1.024	1.015–1.034	<0.001
ST segment deviation > 0.5 mm	0.538	0.435–0.667	<0.001
Year of inclusion	0.913	0.872–0.956	<0.001
Hypertension	1.303	1.051–1.615	0.016
Diabetes	1.419	1.090–1.848	0.009
Previous PCI	1.386	1.042–1.845	0.025
Previous CABG	1.389	0.997–1.936	0.052
Hospitalisation at weekend	1.702	1.345–2.153	<0.001

*CABG* coronary artery bypass graft, *CI* confidence interval, *OR* odds ratio, *PCI* percutaneous coronary intervention

### Outcome

All-cause mortality at 30-day follow-up was 1.3% in the early and 1.4% in the delayed treatment groups (hazard ratio (HR) 1.07, 95% confidence interval (CI) 0.49–2.35, Table 3). This difference was not statistically significant. Recurrent myocardial infarction occurred in 0.8% of both groups (HR 1.02, 95% CI 0.37–2.81). The incidence of total bleeding events was 18.2% in the early and 16.7% (HR 0.89, 95% CI 0.71–1.11) in the delayed treatment group and of non-CABG related bleeding 3.4% and 3.2%, respectively (HR 0.92, 95% CI 0.55–1.53). At 1‑year follow-up, all-cause mortality was significantly higher in the delayed treatment group (7.0 vs 4.1%, HR 1.67, 95% CI 1.12–2.49) but after adjustment for GRACE risk score and other confounding factors, this difference was no longer statistically significant. The prognosis of high-risk patients that did not undergo coronary angiography was significantly worse, with mortality of 20.5% and 42.2% at 30-day and 1‑year follow-up respectively.

**Table 3:** Incidence, HR, 95% CI and *p*-value for outcome parameters in delayed versus early intervention with univariate analysis and adjusted for confounding factors

	Early intervention	Delayed intervention	HR	95% CI	*p*-value
*All-cause mortality 30-day follow-up*					
Incidence (%)	1.3	1.4	1.07	0.49–2.35	0.86
– Adjusted for GRACE risk score			0.68	0.29–1.62	0.39
– Adjusted for serum creatinine, age, ST deviation and previous CABG			0.74	0.31–1.73	0.48
*Reinfarction 30-day follow-up*					
Incidence (%)	0.8	0.8	1.02	0.37–2.81	0.97
– Adjusted for GRACE risk score			1.19	0.40–3.57	0.75
– Adjusted for diabetes, previous MI and age			0.66	0.23–1.88	0.44
*Total bleeding events 30-day follow-up*					
Incidence (%)	18.2	16.7	0.89	0.71–1.11	0.28
– Adjusted for GRACE risk score			0.86	0.67–1.10	0.24
– Adjusted for age, ST deviation, previous CABG, max. CK			0.88	0.69–1.12	0.29
*Non-CABG-related bleeding events 30-day follow-up*					
Incidence (%)	3.4	3.2	0.92	0.55–1.53	0.75
– Adjusted for GRACE risk score			0.73	0.41–1.27	0.26
– Adjusted for age, previous PCI and ST deviation			0.85	0.50–1.43	0.53
*All-cause mortality 1‑year follow-up*					
Incidence (%)	4.1	7.0	1.67	1.12–2.49	0.01
– Adjusted for GRACE risk score			0.71	0.44–1.13	0.15
– Adjusted for age, diabetes, previous CABG			0.83	0.55–1.25	0.37

*CABG* coronary arterial bypass grafting, *CI* confidence interval, *CK* creatine kinase, *GRACE* Global Registry of Acute Coronary Syndromes, *HR* hazard ratio,* MI* myocardial infarction, *PCI* percutaneous coronary intervention

## Discussion

In this prospective registry of 2,299 high-risk NSTE-ACS patients hospitalised between 2006 and 2014, the percentage of patients that underwent coronary angiography increased from 77 to 90% between 2006 and 2014. Concurrently, median time from admission to angiography decreased from 23.3 to 14.5 h with an increase from 50 to 60% of patients being treated within 24 h of admission and fulfilling the criteria of early invasive treatment used in the guidelines. This reflects more stringent adherence to the guidelines concerning the timing of treatment in this patient category.

These changes over time are in line with findings in other registries [12–18]. In general, use of angiography and PCI increased over time, although differences were seen between geographical regions and age groups. This rising trend in adherence to guideline treatment recommendations is associated with improved outcome such as lower mortality and fewer hospitalisations for heart failure [16–18].

Patient factors independently related to delayed coronary angiography in this study were higher GRACE score, higher age and the presence of comorbidities such as hypertension, diabetes and established cardiovascular disease. This inverse relationship between risk profile and the use of invasive treatment has been found in many other observational studies [14, 19–22]. In contrast to the guidelines, which advise an early invasive strategy especially in higher-risk NSTE-ACS patients, this is obviously not applied in daily practice. The GRACE [15, 19], Canadian ACS [14, 20] and CRUSADE [21] registries found that invasive treatment was paradoxically more often applied in lower-risk patients. Waiting time for angiography was also longer in higher-risk patients [22]. Despite the more stringent guidelines concerning the timing of intervention in NSTE-ACS patients, our results are similar to those found in previous studies. Likewise, aggressive anti-thrombotic pharmacotherapy was prescribed more often in low-risk patients in our study, compatible with findings in registries in Canada and the United States [14, 20]. However, the decreased prescription of GPIIb/IIIa receptor blockers in the delayed group could be explained by the lower rate of percutaneous interventions.

A possible explanation for this so-called ‘risk-paradox’ is that cardiologists have more safety concerns with early invasive treatment in older patients presenting with NSTE-ACS. Although invasive treatment is associated with significant benefits independent of age [23–25], age appeared to be underrated as a risk factor in deciding whether or not to perform angiography [26, 27]. However, it is also possible that other valid reasons led to a reluctance to perform invasive procedures in older patients, such as functional status, patient’s preference and frailty, which has been shown to be related to a worse outcome in NSTE-ACS [28].

Patients hospitalised at weekends were more likely to receive angiography after more than 24 h. Although this is a well-known phenomenon, this was not seen in other registries [22, 29] and is probably related to logistics and planning of angiography.

Although patients in the delayed treatment group had a higher risk profile, no differences were found in the incidence of mortality, reinfarction and bleeding at 30-day follow-up, either with univariate analysis and when adjusted for confounding factors. Although it should always be taken into account that unidentified confounders may be present, this suggests that the timing of treatment does not impact on clinical outcome to a great extent. The higher all-cause mortality at 1‑year follow-up in the delayed treatment group was driven by higher age and other risk factors and was no longer present after correction by multivariate logistic regression. Post hoc subgroup analysis of patients with a GRACE risk score >140 showed no significant differences in outcome with early or delayed intervention.

Our study has the following limitations. First of all, it is possible that important predictive factors associated with the timing of intervention have not been taken into account in our study: these include frailty, patient preference and comorbidity. Next, as this was a single-centre study, the generalisability of our findings to high-risk NSTE-ACS patients in general is questionable because local procedures may differ between hospitals and regions. However, similar results were found by previous multicentre studies [14, 19–22]. Also, we excluded patients who were initially hospitalised in non-interventional hospitals to avoid bias by logistic factors. The characteristics of this group of patients might differ from those hospitalised directly at an interventional centre. The outcome results in the delayed treatment group may be too favourable due to survival bias; the effect of patients who die early after hospitalisation is missing. Finally, results of observational, non-randomised studies should always be interpreted with caution because unidentified confounders may be present.

## Conclusions

The percentage of high-risk NSTE-ACS patients undergoing coronary angiography has increased in the last decade, together with a decrease in time from admission to angiography. In contrast to the guidelines, patients with a higher risk profile were less likely to receive an early invasive treatment strategy. However, after adjustment for this higher risk, no difference in outcome after 30 days and 1 year was found.
